# A Comprehensive Genomic Analysis of the Emergent *Klebsiella pneumoniae* ST16 Lineage: Virulence, Antimicrobial Resistance and a Comparison with the Clinically Relevant ST11 Strain

**DOI:** 10.3390/pathogens11121394

**Published:** 2022-11-22

**Authors:** Romário Oliveira de Sales, Laura Leaden, Letícia Busato Migliorini, Patricia Severino

**Affiliations:** Albert Einstein Research and Education Institute, Hospital Israelita Albert Einstein, Sao Paulo 05652-900, Brazil

**Keywords:** *Klebsiella pneumoniae*, ST16, ST11, antimicrobial resistance, virulence factors, high-risk clone

## Abstract

*Klebsiella pneumoniae* is considered an opportunistic pathogen frequently involved with healthcare-associated infections. The genome of *K. pneumoniae* is versatile, harbors diverse virulence factors and easily acquires and exchanges resistance plasmids, facilitating the emergence of new threatening clones. In the last years, ST16 has been described as an emergent, clinically relevant strain, increasingly associated with outbreaks, and carrying virulence factors (such as ICE*Kp*, *iuc*, *rmpADC/2*) and a diversity of resistance genes. However, a far-reaching phylogenetic study of ST16, including geographically, clinically and temporally distributed isolates is not available. In this work, we analyzed all publicly available ST16 *K. pneumoniae* genomes in terms of virulence factors, including capsular lipopolysaccharide and polysaccharide diversity, plasmids and antimicrobial resistance genes. A core genome SNP analysis shows that less than 1% of studied sites were variant sites, with a median pairwise single nucleotide polymorphism difference of 87 SNPs. The number and diversity of antimicrobial resistance genes, but not of virulence-related genes, increased consistently in ST16 strains during the studied period. A genomic comparison between ST16 and the high-risk clone ST11 *K. pneumoniae*, showed great similarities in their capacity to acquire resistance and virulence markers, differing mostly in the great diversity of capsular lipopolysaccharide and polysaccharide types in ST11, in comparison with ST16. While virulence and antimicrobial resistance scores indicated that ST11 might still constitute a more difficult-to-manage strain, results presented here demonstrate the great potential of the ST16 clone becoming critical in public health.

## 1. Introduction

The Gram-negative bacterium, *Klebsiella pneumoniae*, is considered an opportunistic pathogen associated with infections in hospitalized or otherwise immunocompromised individuals [1,2,3]. *K. pneumoniae* contains a core genome, and a large and variable accessory genome harboring virulence factors and antimicrobial resistance genes associated with hospital-acquired infections [2]. Among the most important virulence factors, lipopolysaccharides and polysaccharide capsule that help to evade the immune system, and iron-chelating siderophores, such as yersiniabactin (*ybt*) and colibactin (*clb*), are located on integrative conjugative elements (ICE*Kp*), and aerobactin and salmochelin are plasmid encoded [3,4,5]. Other important virulence mechanisms are associated with the hypermucoviscous phenotype and the presence of the capsular regulator genes rmpA, rmpA2 and magA that up regulate capsule production [2,3,6]. The hypervirulent (hv) phenotype of *K. pneumoniae* is strongly associated with the presence of capsular regulator genes and siderophores [3,7]. The accessory genome is also central to antibiotic resistance in *K. pneumoniae* [2]. Resistance to carbapenems is mainly attributed to the presence of carbapenemases, including *K. pneumoniae* carbapenemase (KPC), Oxacilinases (OXA) and New Delhi metallo-betalactamase (NDM) [3,8,9]. Due to the versatility of acquiring and exchanging resistance plasmids of *K. pneumoniae*, nosocomial infection surveillance is necessary to collect genomic data and identify new and emerging clones [3,10].

Genomic surveillance is being used increasingly to monitor hypervirulence and carbapenemase genes in *K. pneumoniae* isolates [2,3]. The well-characterized high-risk clonal complex (CC), 258 (ST11, ST258, ST512 and ST437), is frequently associated with Klebsiella carbapenemase KPC, and harbors numerous other acquired resistance genes [3,11]. ST11 *K. pneumoniae* is an extensively drug-resistant clone, disseminated worldwide [3,12,13]. The infections caused by XDR *K. pneumoniae* decrease the clinical therapeutic options [12]. The convergence of hypervirulence and the presence of carbapenemase genes have become a threat to public health [2,7]. Recently, a fatal outbreak caused by a hypervirulent carbapenem-resistant ST11 isolate was reported in China; no antibiotics were effective in treating the infections caused by these strains [14].

*K. pneumoniae* genotypes that do not belong to CC258, among which ST16, have emerged as clinically relevant [15,16]. Increased worldwide dissemination, its association with nosocomial outbreaks, as well as increased virulence of ST16 when compared to ST258 or ST11 in *Galleria mellonella* pathogenicity models [15,16,17], suggest that this clone possesses acquired molecular characteristics worth investigation.

Since 2008, numerous hospital surveillance programs from different countries reported ST16 carrying multiple carbapenemases. In Denmark, CTX-M-15 and SHV-1-producing ST16, and ST16 harboring NDM-5 were reported in 2008 and 2015, respectively [18,19]. ST16 associated with CTX-M-15 caused infections in a hospital in France, and in Italy a carbapenem-resistant ST16 clone co-producing NDM-1 and OXA-232 was also reported [20,21]. Furthermore, ST16 carrying NDM-1 was reported in the United Kingdom, Ireland, Taiwan, Croatia, Bulgaria and Canada [22,23,24,25,26,27]. In Vietnam, ST16 isolates harboring OXA-48 or NDM-4 have been reported, and NDM-4 was also observed in Egypt [28,29]. In Brazil, resistance surveillance programs have reported KPC-producing ST16 isolates and isolates harboring OXA-370 [15,30,31,32].

ST16 has become a worrisome clone to public health but a comprehensive phylogenetic study of ST16, including geographically, clinically and temporally diverse isolates is not available. In this work, we provide an in-depth genomic analysis of ST16 isolates and we propose an extensive genomic comparison between ST16 and the well-known high-risk clone ST11. ST11 is a diverse clade from which ST258 derived by recombination. ST11 represents ~12% of carbapenem-resistant *K. pneumoniae* across Europe and is much more broadly distributed than ST258 or ST512.

## 2. Materials and Methods

### 2.1. K. pneumoniae ST16 Genomes

All genome sequences (complete genomes, contigs, scaffolds, or chromosome level genome sequences) of *K. pneumoniae* publicly available at the Genbank (https://www.ncbi.nlm.nih.gov/assembly, accessed on 5 May 2022) were considered for this study. This dataset consisted of 1434 complete genomes, 196 chromosome level genomes, 30,000 contigs and 5782 scaffolds. Associated metadata (date of isolation, host, and geographic location) were collected from NCBI (https://www.ncbi.nlm.nih.gov, accessed on 5 May 2022) when available. All 37,412 selected sequences were submitted to in silico Multilocus Sequence Typing (MLST) (https://github.com/tseemann/mlst, created by Torsten Seemann, Victoria, Melbourne, Australia, accessed on 5 May 2022) for the identification of ST16 isolates. This script implements the Pasteur Institute MLST scheme for *K. pneumoniae* [33]. In total, 2.3% (861/37412) sequences from GenBank were considered ST16 and included in this work.

A second set of 454 *K. pneumoniae* genomes from Pathogenwatch (https://pathogen.watch/, accessed on 10 May 2022) were available in May 2022, and recently described by Argimón et al. [10]. When genomes were found in both Pathogenwatch and Genbank, only the Genbank genome was maintained, and 96 genomes available only in Pathogenwatch were kept for this study.

In summary, 957 ST16 *K. pneumoniae* genome sequences were analyzed in this study.

### 2.2. In Silico K:O Typing

Capsule polysaccharide (K-type) and lipopolysaccharide (O-type) serotypes were determined using Kleborate v2.2.0 (https://github.com/katholt/Kleborate, created by Ryan Wick, Victoria, Melbourne, Australia, accessed on 5 May 2022) using default parameters with confidence levels of “good” or above [34,35].

### 2.3. Resistome, Virulome and Mobilome

The resistome was determined using the NCBI Antimicrobial Resistance Gene Finder Plus (AMRFinderPlus) v.3.10.24 (https://github.com/ncbi/amr, created by Michael Feldgarden, Bethesda, Maryland, United States, accessed on 5 May 2022) with the database “Core” and “Plus”, and the microorganism Klebsiella as previously described [36]. The resistome data was used to evaluate changes in resistance patterns during the studied period. Results are shown in percentages representing the number of AMR genes or AMR gene variants identified per year over the total number of AMR genes or AMR gene variants identified in all ST16 genomes. Additionally, Kleborate v2.2.0 (https://github.com/katholt/Kleborate, created by Ryan Wick, Victoria, Melbourne, Australia, accessed on 5 May 2022) was used to determine the following resistance scores to ST16 isolates: 0 = no ESBL or carbapenemase; 1 = ESBL without carbapenemase (regardless of colistin resistance); 2 = carbapenamase without colistin resistance (regardless of ESBL); 3 = carbapenemase with colistin resistance (regardless of ESBL).

The ABRIcate v.1.0.1 tool (https://github.com/tseemann/abricate/, created by Torsten Seemann, Melbourne, Australia, accessed on 5 May 2022) was used for the identification of virulence genes and plasmid incompatibility groups through the screening of the virulence database VFDB and PlasmidFinder, respectively [37,38]. Genes were considered present if there was 90% coverage at 90% nucleotide identity [14,39].

As a complementary approach, Kleborate v2.2.0 identified integrating conjugative elements (ICE*Kp*), iuc (aerobactin), iro (salmochelin), the genotoxin locus clb (colibactin), and plasmid-associated virulence loci [34]. Virulence scores were assigned using Kleborate v2.2.0 (https://github.com/katholt/Kleborate, created by Ryan Wick, Victoria, Melbourne, Australia, accessed on 5 May 2022) based on the presence of ybt, clb and iuc (Score 0 = none present; 1 = ybt only; 2 = clb without iuc (regardless of ybt;); 3 = iuc only; 4 = iuc and ybt without clb; and 5 = all three genes present).

Mobile Genetic Elements (MGEs) were identified using MobileElementFinder v1.0.5 default parameters available at (https://pypi.org/project/MobileElementFinder/, created by Markus Johansson, Lyngby, Denmark, accessed on 5 May 2022) [40].

Finally, AMR-virulence convergence in ST16 was analyzed based on resistance and virulence scores (convergence defined as virulence score ≥3 and resistance score ≥1) generated using Kleborate as described by Lam et al. [15].

### 2.4. Pan Genome Analysis

All 957 ST16 *K. pneumoniae* genomes were annotated using prokaryotic genome annotation pipeline, with default parameters Prokka v1.14.6 [41]. Roary v3.13.0 was used to generate the pan genome profile with a 95% BLAST v2.10.1 identity threshold using the MAFFT v7.475 (https://mafft.cbrc.jp/alignment/software/, created by Kazutaka Katoh, Suita, Osaka, Japan, accessed on 5 May 2022) setting. The core genome was defined as “genes present in at least 99% of genomes” using the 957 gff3 files obtained with Prokka v1.14.6 as previuously described [42]. Recombination sequences were identified and removed from the global core genome alignment with fastGEAR software v.1.0 (https://users.ics.aalto.fi/~pemartti/fastGEAR/, created by Rafal Mostowy, London, United Kingdom, accessed on 5 May 2022) [43,44]. Snp-dists (https://github.com/tseemann/snp-dists, created by Torsten Seemann, Melbourne, Australia, accessed on 5 May 2022) was used to create a pairwise SNP distance matrix from the core gene alignment. The median nucleotide divergence of core genes analyzed was calculated by taking median pairwise SNP differences and dividing by total length of the core genome minus ambiguous characters [45]. IQ-TREE-v2.2.0 (http://www.iqtree.org/, created by Bui Quang Minh, Vienna, Austria, accessed on 5 May 2022) was used to create a Maximum likelihood based on the core genome defined using Roary [46]. ModelFinder identified the best nucleotide substitution (GTR+F+I+I+R5 model with a rate heterogeneity ‘FreeRate’ (http://www.iqtree.org/, created by Subha Kalyaanamoorthy, Edmonton, Alberta, Canada, accessed on 5 May 2022) [47]. Bootstrapping was done 1000 times with ultrafast bootstrap (UFBoot) (http://www.iqtree.org/, created by Diep Thi Hoang, Hanoi, Vietnam, accessed on 5 May 2022). The tree was visualized using Microreact v.218 (https://microreact.org/, created by Silvia Argimón, Hinxton CB10 1SA, United Kingdom, accessed on 5 May 2022) [48].

### 2.5. Genome Comparison of ST16 and ST11 K. pneumoniae

Due to the clinical importance and worldwide spread of ST11, we compared the genomic characteristics of ST11 and ST16 *K. pneumoniae* genomes. The 1247 ST11 genomes recently analyzed by Argimón et al. [10] (ciab784_suppl_Supplementary_Table_3 and ciab784_suppl_Supplementary_Table_6) and all ST16 genomes selected in this study were used for these comparisons.

## 3. Results

### 3.1. Characterization of ST16 Dataset

**Figure 1:**
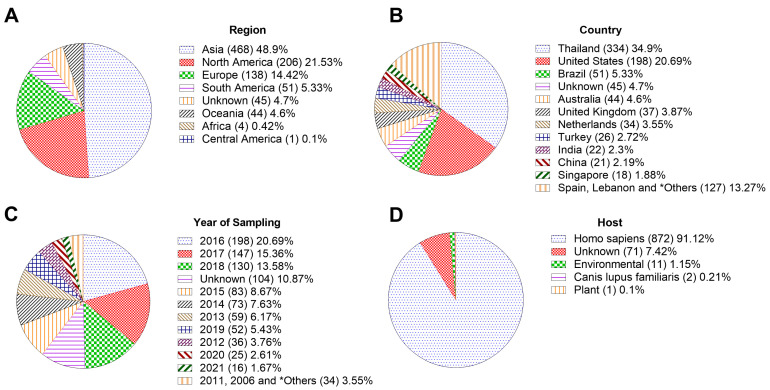
Diversity of *K. pneumoniae* ST16 isolates included in this study. (**A**) Distribution by geographic region. (**B**) Distribution by country of the available genomes. (**C**) Temporal distribution of isolates. * Others: comprise isolates reported between 2003 and 2022. (**D**) Hosts or sites of sampling.

ST16 isolates analyzed in this study were mostly collected from human hosts over a period of 19 years (2003 to 2022) worldwide: Asia (468/957; 48.9%), North America (206/957; 21.53%), Europe (138/957; 14.42%), South America (51/957; 5.33%), Oceania (44/957; 4.6%), Africa (4/957; 0.42%) and Central America (1/957; 0.1%) (Figure 1).

### 3.2. Antimicrobial Resistance (AMR) Genes in ST16 K. pneumoniae Genomes

AMR genes identified in ST16 isolates were associated with resistance to the following 13 classes of antimicrobials: aminoglycoside, beta-lactam, colistin, quinolone, bleomycin, fosfomycin, macrolide, phenicol, rifamycin, streptothricin, sulfonamide, tetracycline and trimethoprim (Table S1). Most ST16 isolates carried genes associated with carbapenem resistance (734/957; 76.69%), extended-spectrum beta-lactamase (ESBL) genes (956/957; 99.89%), or both (733/957; 76.59%). Few isolates carried no genes associated with carbapenem resistance (223/957; 23.30%), and only one isolate carried no ESBL gene (1/957; 0.10%).

Currently, *K. pneumoniae* shows high resistance to beta-lactam antibiotics, fluoroquinolones and aminoglycosides [49,50,51], and mobilized colistin resistance (MCR) genes are becoming an important health care threat [52]. For these reasons, we highlight AMR genes conferring resistance to these four classes of antimicrobials. Thirty one different genes associated with aminoglycoside susceptibility were identified, with aadA2 (693/957; 72.41%), aac(6′)-Ib-cr5 (419/957; 43.78%), aac(6′)-Ib (364/957; 38.03%), aadA1 (328/957; 34.27%) and aph(3′)-Ia (225/957; 23.51%) being the most commonly found. Noteworthy was the presence of genes related to enzymatic target site modification by 16S rRNA methyltransferases (RMTs), rmtB1 (77/957; 8.04%) and rmtF1 (50/957; 5.22%), and the aminoglycoside-modifying enzyme armA (16/957; 1.67%), leading to higher drug resistance levels [53]. Concerning resistance to β-lactam antibiotics, including ESBL, 48 different genes were identified. The *bla*_SHV-1_ gene was the most prevalent ESBL gene (919/957; 96.02%), followed by *bla*_CTX-M-15_ (821/957; 85.78%) and *bla*_TEM-1_ (646/957; 67.50%).

*K. pneumoniae* carbapenemase (KPC) encoding genes were identified in 11.59% of the isolates *(n* = 111), with *bla*_KPC-2_ (52/957; 5.43%) and *bla*_KPC-3_ (43/957; 4.49%) being the most common variants. Considering prevalence and geographical distribution, *bla*_KPC-2_ was mostly reported in Brazil (31/52; 59.61%), and *bla*_KPC-3_ was mainly found in the United States (41/43, 95.34%). Both countries accounted for 78.37% (87/111) of KPC encoding genes reported for ST16.

The New Delhi metallo-β-lactamase 1 (NDM-1) was identified in 37.90% of the isolates (334/957), most of which were from Thailand (275/334; 88.33%). The *bla*_OXA-1_ β-lactamase gene (class D) was detected in 40.33% (386/957) of the isolates. Additionally, 8.35% (80/957) of the 957 genomes carried the *bla*_OXA−48_ gene, mostly reported in the Netherlands (19/80; 23.75%), Singapore (13/80; 16.25%), Spain (13/80; 16.25%) and Turkey (11/80; 13.75%). Other carbapenemase encoding genes, such as *bla*_KPC-14_, *bla*_NDM-4_, *bla*_NDM-5_, *bla*_NDM-7_, *bla*_VIM-1_, *bla*_IMP-1_ and *bla*_IMP-6_ were also detected (Table S1).

Fluoroquinolone resistance genes belonging to the core genome were identified in almost all ST16 *K. pneumoniae* genomes: *oqxA* (949/957; 99.16%) and *oqxB32* (916/957; 95.71%) [54]. However, acquired resistance to fluoroquinolone was represented mostly by *qnrS1* (97/957; 10.13%), *qnrB1* (45/957; 4.70%) and *qnrB6* (36/957; 3.76%). These genes may be carried by plasmids [55,56,57].

Finally, MCR encoding genes were identified in 0.73% (7/957) of ST16 *K. pneumoniae* genomes and reported only in Asia and Europe: the *mcr-1* gene in Thailand (4/5; 80%) and Vietnam (1/5; 20%), and *mcr-9* in Romania (2/2; 100%).

**Figure 2:**
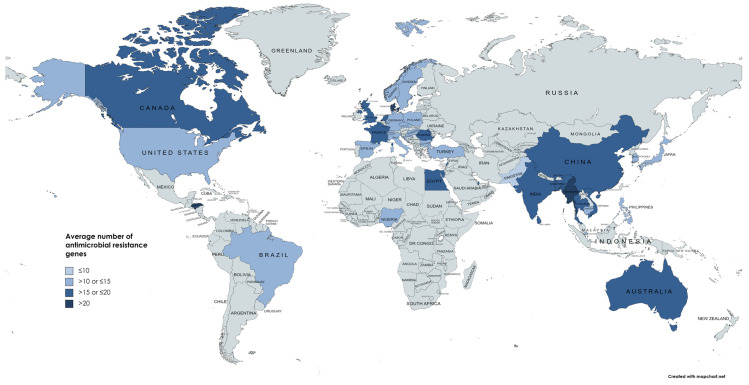
Heatmap illustrating the number of antimicrobial resistance genes per location. Gray: location with no ST16 isolates reported in NCBI. This figure was made using MapChart (https://www.mapchart.net/world.html; accessed on 5 May 2022) and data from 912 isolates for which sampling location was available.

**Figure 3:**
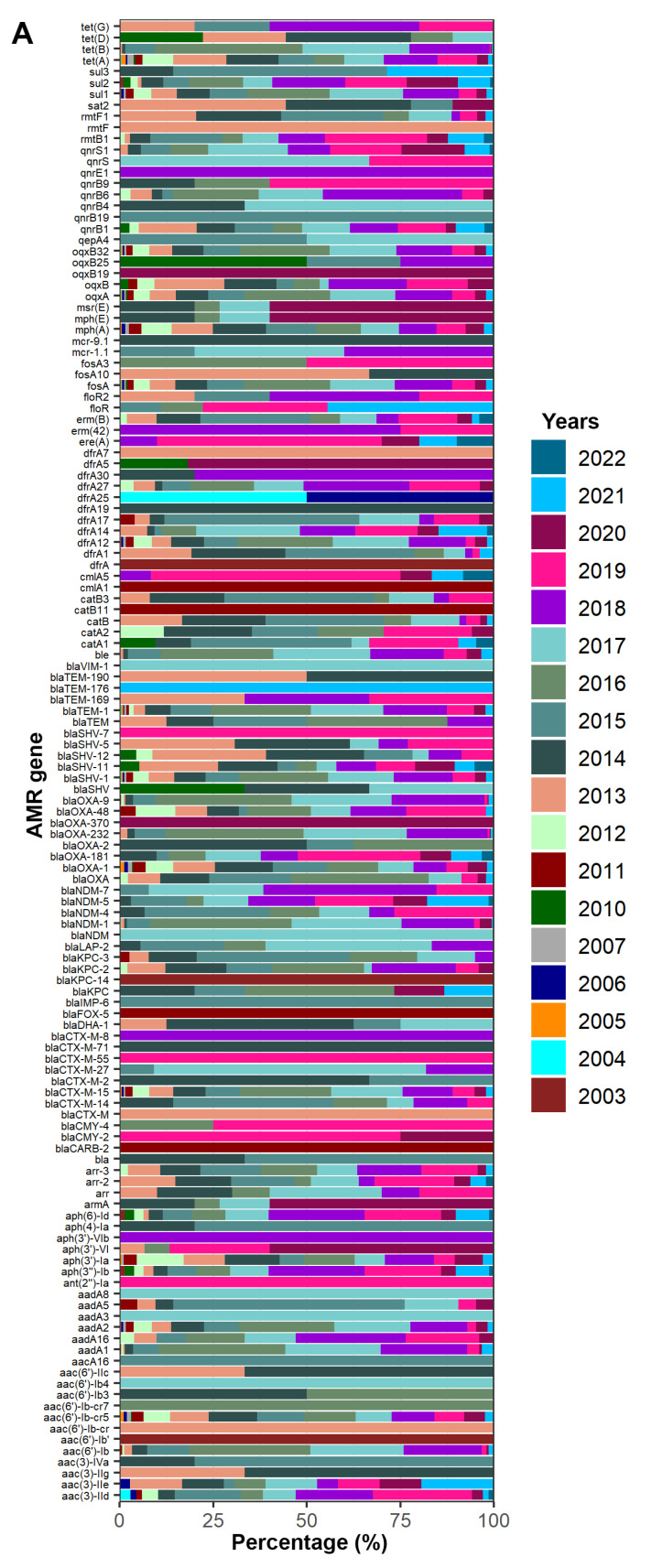

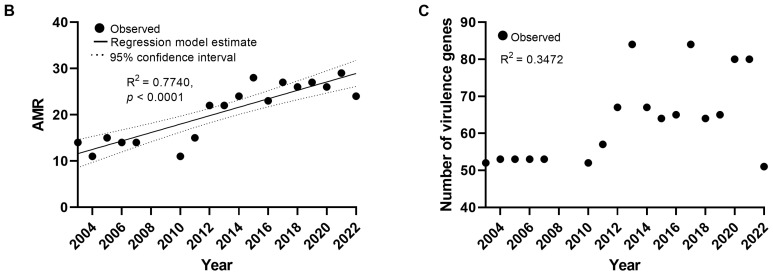
(**A**) Distribution of 132 AMR genes reported in 853 genomes of *K. pneumoniae* ST16. (**B**) Distribution of the maximum number of AMR genes or AMR gene variants per genome per year. (**C**) Distribution of the maximum number of virulence genes per genome per year.

In order to understand how AMR genes are distributed in ST16 isolates worldwide, and if resistance patterns changed in time, we analyzed 912 isolates for which information about the location for sampling was available. A heatmap illustrating the number of antimicrobial resistance genes found per location is presented in Figure 2. According to reported data, the average of number of AMR genes was lower in Pakistan and Hong Kong (≤10) and higher numbers were found in Myanmar, Denmark and Honduras (>20) (Figure 2). For a total of 853 genomes (89.13%) the year of isolation was available, the first ST16 genome having been reported in 2003 and the last one in 2022. A diverse set of AMR genes (Figure 3A) and a significant increase in maximum AMR gene numbers per genome per year were observed (*p* < 0.0001, R^2^ = 0.7740) (Figure 3B). On the other hand, the maximum number of virulence genes per genome did not increase over time (R^2^ = 0.3472) (Figure 3C).

### 3.3. Plasmid Incompatibility Groups and Transposons

**Figure 4:**
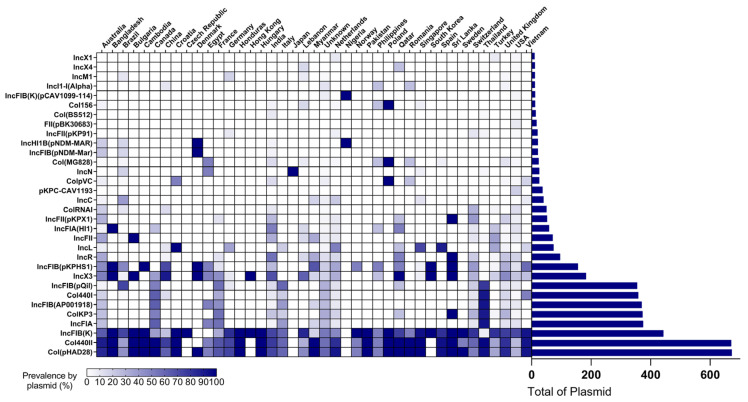
Plasmid replicon screening. PlasmidFinder was used to screen the genomes of ST16 *K. pneumoniae*. Only plasmid replicons found in ≥10 isolates are reported in this figure.

Fifty-nine different plasmid incompatibility groups were detected, with almost 100% of isolates harboring at least one of such structures (949 out of 957 isolates). The most common Inc groups were Col(pHAD28) (673/949; 70.91%), Col440II (971/949; 70.70%), IncFIB(K) (443/949; 46.68%), IncFIA (375/949; 39.51%), ColKP3 (373/949; 39.30%), IncFIB(AP001918) (370/949; 39.98%), Col440I (359/949; 37.82%) and IncFIB(pQil) (355/949; 37.40%) (Figure 4).

Tn*4401* is the most common transposon associated with KPC encoding genes [58,59]. In ST16 isolates harboring KPC encoding genes (111/957; 11.59%) transposons Tn*4401*-like was found in almost every isolate (106/111; 95.49%). NTE (non-Tn*4401*) group NTEKPC-Inc were found in two isolates, and the other three genomes harbored structures that could not be characterized (Tn3-Tn3-IS481(ISKpn7)-KPC-ISKpn6 (*n* = 1), KPC-IS1182 family transposase (*n* = 1), and ISKpn6-KPC-IS21-IS21 (*n* = 1).

### 3.4. Virulence Factors and Heavy Metal Resistance

Capsular polysaccharide (K antigen) and lipopolysaccharide (O antigen) are important virulence factors in *K. pneumoniae*, with clinical and epidemiological importance including the identification of hypervirulent strains [54,60]. More than 138 combinations of K-locus are currently known [54]. In this study, 10 different K-types were identified (Table S2), with KL51 being the most prevalent (759/957; 79.31%). Six different O-types were found, with O3b as the most prevalent (886/957; 92.58%) (Table S2).

**Figure 5:**
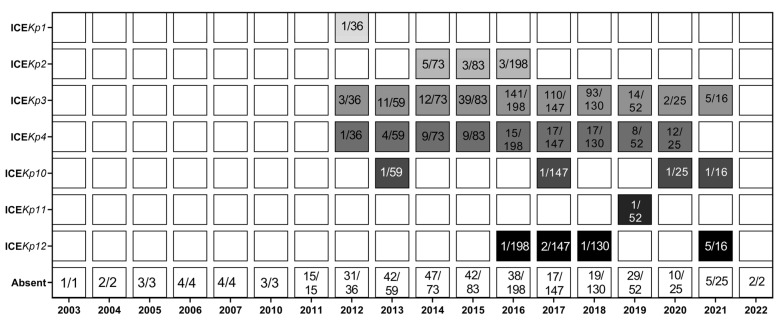
Prevalence of Integrative Conjugative Elements de *K. pneumoniae* (ICE*Kp*).

Chromosomally inserted ICE*Kp* with an associated yersiniabactin gene (*ybt*) cluster, known to contribute to hypervirulence [4,45], was found in 63.21% (605/957) of the isolates (Table S2). Seven distinct types of ICE*Kp* were identified (Figure 5), the most commonly found ICE*Kp* was ICE*Kp*3-*ybt*9 (460/605; 76.03%), followed by ICE*Kp*4-*ybt*10 (119/605; 19.66%). In this dataset ICE*Kp* was first identified in an isolate from 2012 (Figure 5). ICE*Kp* was mostly identified in isolates from Thailand, between 2014 and 2018, corresponding to 54.04% (327/605) ICE*Kp*-harboring isolates. Even though this country contributed with 34.90% (334/957) of the isolates included in this study, this high prevalence is very different from results from the United States which contributed with the second highest number of isolates (198/957); only 4.70% (45/198) of isolates from this country harbored ICE*Kp*.

A combination of ICE*Kp*10 and of colibactin (*clb*3), which induces DNA damage in eukaryotic cells [54], was detected in four genomes, two of them isolated in the United States and two in Brazil. Besides *clb*3, other markers of hypervirulence were detected in ST16 genomes: *rmpADC* and/or *rmpA2* in 11 genomes (1.14%), aerobactin in 26 genomes (2.71%), and salmochelin in 2 genomes (0.20%).

AMR and virulence genes used to be segregated in non-overlapping *K. pneumoniae* populations, but recent reports highlight the convergence of AMR-virulent strains which have the potential to cause difficult-to-treat infections [34]. In 25 genomes evaluated in this study, AMR-virulence convergence was detected, a result that highlights the need to monitor the dissemination of ST16, especially in Australia, India and the United States, where these isolates were reported.

Resistance to heavy metal may contribute to the selection and dissemination of MDR strain [61]. Genes associated with heavy metal resistance were identified in 50.47% of isolates (483/957) (Table S3), the most prevalent ones being operon *pcoABCDERS*, associated with resistance to copper [62], detected in 44.30% of isolates (424/957), and *silABCEFPRS*, associated with resistance to silver [63], and detected in 43.57% of isolates (417/957). Other important findings were: genes associated with resistance to arsenic (436/957; 45.55%) including arsenical pump-driving ATPase (*arsA*), and *arsB*, *arsC*, *arsD*, *arsR* [64], resistance to mercury (mer) (81/957; 8.46%) [65], tellurium resistance (*ter*) (25/957; 2.61%) and nickel resistance (*ncrABY*) (13/957; 1.35%). Despite the fact that Thailand contributed with the highest number of genomes to this study (334/957; 34.90%), only 6.88% (23/334) of them carried genes associated with resistance to heavy metal; contrasting with the United States, the second country in number of isolates (198), of which 80.80% (160/198) harbored at least 1 gene associated with resistance to heavy metal.

### 3.5. Genetic Diversity of ST16 Genomes

Considering the 957 ST16 isolates analyzed in this study, the pan genome consisted of 29,510 genes, of which 3761 (12.74%) made up the core genome (i.e., genes found in 99% of full genomes). The accessory genome, 25,749 genes, consisted of 23,601 uncommon genes, present in ≤15% of the isolates (cloud genes) and 1495 shell genes, present in <95% of the isolates.

Normalized polymorphism counts were used as a measure of genetic variation within the population, and we found the overall species median nucleotide divergence to be 0.003356% (median pairwise single nucleotide polymorphism [SNP] difference = 87 SNPs), suggesting that less than one percent of the core genome nucleotide sites were variant sites. This nucleotide diversity is comparable to previously shown measures of genetic variation of core genome alignment of *K. pneumoniae* clonal group CG23 [66].

**Figure 6:**
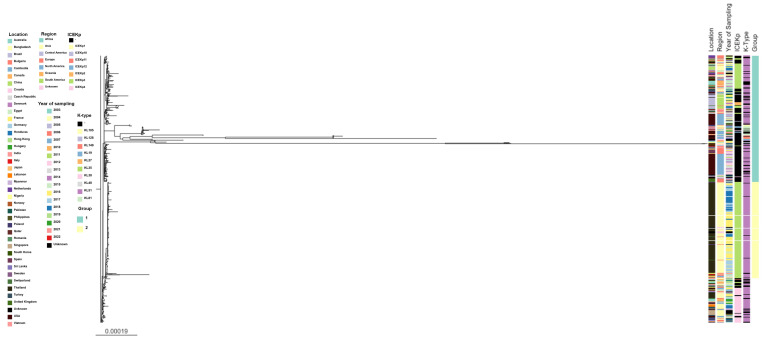
Maximum-likelihood phylogeny of the 957 *Klebsiella pneumoniae* ST16 was inferred from aligned core genomes by roary. (-) ICE*Kp* or K-type were not identified based on the threshold defined in methods. Data available for interactive viewing are available at https://microreact.org/project/drXbkjrfrVUP6TiEQAVh7r-tree-phylogenetic-kp-st16, accessed on 5 May 2022. The greater diversity of ICE*Kp* within group 1 reflects the geographic diversity of this group (37 countries). Almost all isolates from group 2 harbored ICE*Kp*3 (343/344; 99.70%) (Figure 6, which is also available for interactive viewing at https://microreact.org/project/kFHeSw2Qr9cecxdgX7oSnt-tree-phylogenetic-kp-st16-group-2, accessed on 5 May 2022), with the exception of one isolate, also from Thailand, reported in 2017. Despite the widespread of ICE*Kp*3 within this group, 111/452 (24.55%) of isolates from group 1 also harbored ICE*Kp*3.

In order to determine the phylogeny of the 957 ST16 isolates, a maximum-likelihood tree was inferred from the aligned core genomes by roary (Figure 6, which is also available for interactive viewing at https://microreact.org/project/drXbkjrfrVUP6TiEQAVh7r-tree-phylogenetic-kp-st16, accessed on 5 May 2022. Two major groups were identified, one consisting of 47.23% (452/957) of the isolates (Figure 6, which is also available for interactive viewing at https://microreact.org/project/56YFd6ALYc1eLHMzjX6wJa-tree-phylogenetic-kp-st16-group-1, accessed on 5 May 2022), and one by 35.94% (344/957) (Figure 6, which is also available for interactive viewing at https://microreact.org/project/kFHeSw2Qr9cecxdgX7oSnt-tree-phylogenetic-kp-st16-group-2, accessed on 5 May 2022). The first group included isolates from 37 of the 40 countries that reported ST16 isolates, contrasting with the second group that depicted isolates from only 8 countries. The majority of samples from the second group originated from Thailand (89.53% [308/344]), corresponding to 92.21% (308/334) of the genomes reported by this country.

ICE*Kp* was detected in 30.75% (139/452) of isolates from group 1, where the most diversity of ICE*Kp* was also found (ICE*Kp* 1, 2, 3, 4, 10, and 12); only ICE*Kp*11 was not found in this group (Figure 6, which is also available for interactive viewing at https://microreact.org/project/qrmdyoehcemjUDndtbnE4A-tree-phylogenetic-kp-st16-icekp-type, accessed on 5 May 2022), but ICE*Kp*11 was identified in a single isolate from Australia (2019), and this isolate did not cluster in either of the two groups.

Due to the over-representation of isolates from Thailand in group 2 (92.21%, 308/334 isolates) we investigated their origin in more detail. The genomes derive from 8 distinct NCBI Bioprojects (PRJNA389557, PRJNA414481, PRJNA497260, PRJNA532291, PRJNA717739, PRJDB5929, PRJNA644880 and PRJEB19226), 75% of them were reported in Bangkok between 2015 and 2018. The second largest dataset came from the province of Surat Thani (10.38%, 32/308 isolates). Taken together, the data suggest that isolates from Thailand do not correspond to a restricted location and represent the diversity of ST16 found in the country.

A total of 161 isolates did not cluster within groups 1 or 2. Interestingly, 72.04% (116/161) of these isolates carried ICE*Kp*4, and this structure was only found in three isolates (3/452) belonging to group 1. Even though all these 116 isolates harbored ICE*Kp*4, the isolates were significantly distinct in their core genome, distributed in 5 continents (Asia, Central America, Europe, North America and Oceania), and found in a total of 18 countries between 2012 and 2020.

### 3.6. Major Similarities and Differences between ST16 and ST11

Carbapenem resistance among ST11 isolates is usually associated with the dissemination of the *bla*_KPC-2_ gene [10,14,32,67,68]. Here, ST16 isolates were also found to carry the *bla*_KPC_ (111/957; 11.59%), with *bla*_KPC-2_ (52/957; 5.43%) and *bla*_KPC-3_ (43/957; 4.49%) being the most frequently found variants. However, *bla*_NDM-1_ was the most prevalent variant among ST16 isolates (334/957; 37.90%) (Table 1), differently from previous reports for ST11 (4.67–15.1%) [10,14].

The most common gene encoding a carbapenemase among ST16 isolates was *bla*_OXA-232_, present in 38.24% of ST16 isolates (Table 1). The *bla*_OXA-232_ gene was found in ST16 isolates from 13 countries (Thailand, Unknown, Australia, UK, USA, Netherlands, France, Canada, India, Switzerland, Italy, Qatar, Sri Lanka and Turkey), but most frequently found in isolates from Thailand (81.96%) (300 out of 366 isolates from Thailand carried this gene). Due to the high contribution of isolates from Thailand, we reevaluated the data considering only isolates from the other 12 locations, finding that 10.04% of the remaining ST16 isolates (66/657) carried *bla*_OXA-232_. Argimón et al. [10] found *bla*_OXA-232_ in 2.16% (27/1247) of the evaluated ST11 isolates, mostly reported in Oman (18/27; 66.66%), Thailand (8/27; 29.62%) and India (1/27; 3.70%). 

CTX-M-15 is the most prevalent ESBL type in Europe, having also been found in North America and Asia more recently [14]. This gene was detected in 85.78% of the ST16 isolates (Table 1), against 12.08 to 41.9% reported for ST11 [10,14]. On the other hand, *bla*_CTX-M-65_ is prevalent in ST11 isolates (28.9 to 59.61%) [10,14], but not found in ST16 isolates (Table S1). OXA-48 was similarly found in both STs, in 12.5% of ST11 [10] and 8.35% of ST16 (Table 1). In our study, *bla*_OXA-1_ was found in 40.33% ST16 (Table S1), against 12.08% reported for ST11 [14].

CPS is a key virulence determinant in *K. pneumoniae* [14,69]. Twenty-eight different K-types have been described for ST11 isolates (Table 1), with four predominant K-types (KL105, KL24, KL47 and KL64) [10]. This characteristic contrasts with a single K-type (KL51) seen in 80% of ST16 isolates (Table 1). Similarly, three major O-types (O2v1, O2v2, OL101) have been described for ST11 [10], while over 90% of O-types in ST16 were O3b (Table 1).

Yersiniabactin was one of the most common virulence-associated factors detected in both strains, 83.80% in ST11 [10] and 63.21% for ST16, both also sharing ICE*Kp*3 e ICE*Kp*4 as the most prevalent (Table 1). Aerobactin, salmochelin, *rmpADC* and *rmpA2* were found in isolates reported in China: aerobactin (*n* = 35), salmochelin (*n* = 1), *rmpADC* (*n* = 10) and *rmpA2* (*n* = 31) [10]. These markers are, however, more widespread among ST16 isolates: aerobactin (*n* = 26) reported in Australia (*n* = 9), United States (*n* = 16) and India (*n* = 1); salmochelin (*n* = 2) reported in the United States (*n* = 1) and one of unknown origin (*n* = 1); *rmpADC* (*n* = 10) reported in Australia (*n* = 9) and the United States (*n* = 1); and *rmpA2* (*n* = 6) reported in Australia (*n* = 5) and India (*n* = 1) (Table S2).

**Figure 7:**
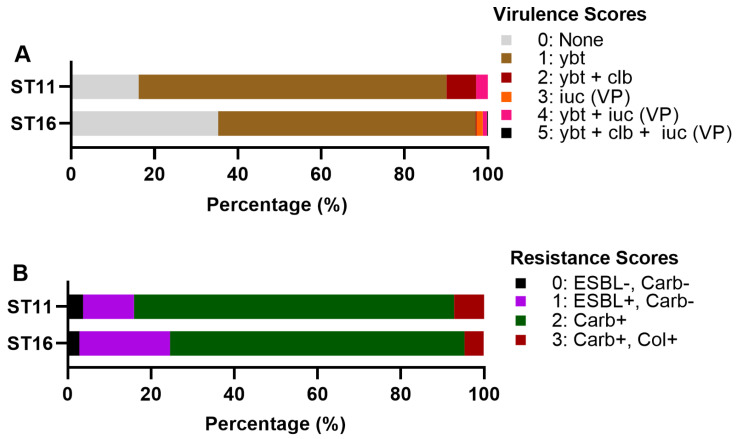
Distribution of (**A**) virulence and (**B**) resistance scores among genomes belonging to ST11 and ST16. Ybt: yersiniabactin, clb: colibactin, iuc: aerobactin, VP: virulence plasmid, ESBL: Extended Spectrum-β-Lactamase, Carb: carbapenemase, Col: colistin resistance determinant.

Kleborate virulence scores capture the hierarchy of virulence-associated loci that have emerged over the last two decades according to the literature [34]. The score considers the presence of *ybt*, *clb* and *iuc*, and is interpreted as follows: score 0 = none present, 1 = yersiniabactin only, 2 = colibactin without aerobactin (regardless of yersiniabactin, however, *ybt* is almost always present when *clb* is), 3 = aerobactin only, 4 = aerobactin and yersiniabactin without colibactin, and 5 = all three present. Yersiniabactin facilitates immune escape and has been shown to increase virulence in various strains [34,70]. The presence of the genotoxin *clb* together with *ybt* may increase virulence due to *clb’s* genotoxic activity in mammalian cells [34,71], but limited evidence indicates increased virulence in the absence of the virulence plasmid [34]. The *iuc* is related to sepsis since it promotes bacterial growth in blood via the acquisition of iron from transferrin [34,72,73]. Taken together, there is limited data assessing the individual contributions of *ybt*, *clb* and the virulence plasmid when present in combination, but it is logical to score *iuc* + *ybt* with higher virulence than *iuc* without *ybt* [34]. The combination of *iuc* (virulence plasmid) + *ybt* + *clb* as the highest score, is associated with hypervirulent strains in liver abscesses documented worldwide (CG23-I) [34]. Kleborate showed similar results for ST11 and ST16, although more ST11 isolates showed Score 2 (7.1%) and isolates showed Score 4 (2.8%) (Figure 7A).

The resistome of ST16 is also alarmingly similar to ST11, as confirmed by the Kleborate score (Figure 7B), although more ST16 isolates belonged to Score 1 (ESBL+ and Carb-) (see Section 2.“Material and Methods”) when compared to ST11.

Kleborate resistance scores do not include the presence of intrinsic variants of oqxAB, chromosomal fosA and ampH since they are not associated with clinical resistance in *K. pneumoniae* species complex (KpSC) [34].

## 4. Discussion

*K. pneumoniae* belonging to ST16 is an important emergent lineage that can carry determinants to carbapenem resistance and is believed to have an enhanced virulence potential, as observed by patient survival analysis and in vitro studies [15,16,74].

According to the core SNP analysis of 957 ST16 genomes, two major groups comprised over 80% of all reported isolates (796/957; 83.17%), with the major group including globally distributed isolates reported in Africa, Asia, Europe, North America, South America and Oceania.

This study showed a significant increase in the maximum number of AMR genes in a genome per year (Figure 3B; *p* < 0.0001), a finding that highlights the importance of monitoring the emergence of extremely drug-resistant (XDR) ST16 isolates, already reported in several locations with distinct antimicrobial resistance profiles and clear evidence of horizontal gene transfers [19,20,75,76,77]. Forty-eight different AMR genes associated with resistance to β-lactam antibiotics, including ESBL, were identified, with a drastic increase in the number of AMR genes per genome since 2012: between 2003 and 2011, four to eight AMR genes or gene variants were found; and between 2012 and 2021, 11 to 28 AMR genes or variants associated with resistance to β-lactam antibiotics, including ESBL, were identified. These finding demonstrate that ST16 isolates have been acquiring different AMR genes and that many of these genes are now widespread among ST16 isolates. The increasing incidence of ESBL and carbapenem resistant of ST16 isolates, coupled with their increasing global distribution, also emphasizes their potential contribution for rapid dissemination of AMR genes to other highly-virulent pathogens [78].

A similar scenario was observed for aminoglycosides, with numbers between 2003 and 2011 varying from two to six genes or variants, and between 2012 and 2021 increasing to 10-18. Thirty-one genes associated with this type of resistance were found, the most common being aac(6′)-Ib-cr5 encoding an aminoglycoside acetyltransferase that also has activity against fluoroquinolones [79,80]. The aac(3)-IVa (*n* = 10 genomes) and aph(4)-Ia (*n* = 10 genomes) were identified exclusively in the United States. For quinolones, there was no significant increase in the number of AMR in time, with two to nine genes or variants identified per genome per year during the studied period. Noteworthy was the presence of qnrE1, reported in five isolates from Brazil in 2018. This gene was originally found in a conjugative plasmid in a clinical *K. pneumoniae* isolate [81]. Also noteworthy are reports of qnrB1 since 2012 in 13 countries but mostly prevalent in the United States (18/45; 40%), and qnrS1, reported since 2013 in 19 different countries but most prevalent in the United States, Australia and Thailand (19/97; 19.58%), (17/97; 17.52%) and (14/97; 14.43%), respectively. Both genes have been found in plasmids [55,56].

The contribution of horizontal gene transfer to the spread of AMR genes could be clearly illustrated by the widespread distribution of ICE*Kp* and plasmids. Seven types of ICE*Kp* (1, 2, 3, 4, 10, 11 and 12) were identified, found in 63.21% (605/957) of the evaluated isolates, similar to a previous report that found ICE*Kp* in 61.04% of the evaluated ST16 genomes [10].

A direct link between AMR genes and plasmids is not always possible with short-read assemblies, but their central role in the dissemination of AMR genes, and importance for epidemiological discussions, is well known [10,82,83]. The two plasmid replicons were widespread in the United States and identified exclusively in this country: FII(pBK30683) (*n* = 17) and pKPC-CAV1193 (*n* = 37). The analysis of 334 ST16 isolates from Thailand identified replicons ColKP3 and IncFIA/B, present in 90.11% (301/334) and 92.21% (308/334) of isolates, respectively, corroborating with findings by Abe et al. [84]. These replicons have been associated with the dissemination of *bla*_NDM-1_ and *bla*_OXA-232_ in Thailand and Canada [84], and ColKP3-harboring *bla*_OXA-232_ was also reported by Argimón et al. [10,85]. *K. pneumoniae* ST16 co-carrying *bla*_NDM-1_ and *bla*_OXA-232_ is considered a successful clone in Thailand, a characteristic they fund to be exclusive to ST16 isolates among 43 STs classifying 577 carbapenem-resistant *K. pneumoniae* isolates in a nationwide surveillance study carried out in the country [86]. Here, we found 286 isolates carrying this combination of genes, 90.20% of which (258/286) were from Thailand.

KPC-encoding genes are often found in transposons, with Tn*4401* being the most prominent transposon type associated with KPC-2 [58]. Accordingly, 95.49% (106/111) of isolates that carried KPC in this study also harbored a Tn*4401*-like transposon. KPC was mostly detected in Brazil and the United States, which together accounted for 78.37% (87/111) of cases. The first report of an ST16 isolate in Brazil harboring KPC-2 occurred in 2008 [30]. Since then, this clone has been often associated with KPC-2, nowadays endemic both in Brazil and the United States [15,16,32,68,87]. The inconsistent carriage of KPC-2 within the ST16 clone suggests the KPC enzyme was acquired through the horizontal transfer of plasmids after the divergence of the ST16 lineage rather than vertical gene transfer among the entire lineage [60].

Differently from AMR genes, the number of virulence genes detected per genome did not increase in the studied period (Figure 3C; R squared = 0.3472). Numerous genetic factors contribute to the ability of *K. pneumoniae* strains to cause disease in humans [54], and we found genomic features that may provide ST16 with an advantage for adaptation within a hospital environment: (1) the majority of ST16 isolates harbored wzi50 (887/957; 92.68%), a characteristic associated with increased virulence [74], (2) ST16 shared various virulence-determinant genes (*ybt-irp-fyuA*, *entA-F*, *fimA-H*, *mrkA-J*, *clb3* and KL51 type for CPS), (3) hypervirulence markers such as aerobactin, salmochelin, *rmpADC* and *rmpA2*, and (4) the locus *ybt* within ICE*Kp*, a virulence factor that directly influences the pathogenicity of *K. pneumoniae* [4,34,70].

*K. pneumoniae* isolates may belong to high-risk clonal groups: hyper-virulent clonal complexes (CCs), mostly responsible for community-acquired invasive infections, and multidrug-resistant CCs, mostly involved in health-care associated infections [60,88,89]. The majority of ST16 isolates investigated here belong to multidrug-resistant CCs, in agreement with previous reports [17,19,76]. Worth noting is that recent studies have shown increased mortality associated with ST16 but have not found specific characteristics of ST16 that justified this association [15,17]. Our results corroborate with these reports since less than 3% of ST16 harbored markers of hyper-virulence, highlighting the need for a deeper understanding of genetic features of ST16.

Finally, due to the clinical importance of *K. pneumoniae* clone ST11 [14,34], we investigated similarities and differences between ST16 and ST11. CPS and LPS O antigen is a key virulence determinant in *K. pneumoniae*. The great diversity of CPS and adjacent LPS O antigen of ST11 is associated with a recombination hotspot at the biosynthesis loci [29,90,91]. In ST16 strains, this hotspot in the biosynthesis loci has not been described, and the ST16 isolates investigated here showed a reduced variability of CPS and LPS, withKL51 and O3b the predominant serotypes.

Although ST11 and ST16 showed similar virulence scores, ST11 has been more studied and associated with hyper-virulence, such as the ST11-KL64 strain reported in China, carrying r*mpA*/*rmpA2*, *iucABCD* and *iutA* genes [29]. Given the global success of ST11 and similarities with ST16 in terms of virulence and antibiotic resistance, this emerging clone has the potential to become a global epidemic dual-risk clone and a major public health threat.

## Figures and Tables

**Table 1:** Main characteristics observed between *Klebsiella pneumoniae* ST11 and ST16.

ST	No. of Genomes in Public Collection	Total No. of Countries	Region Most Prevalent Genomes ≥10% Isolates)	Countries Most Prevalent Genomes ≥10% Isolates)	Total No. of K-Types (and Types With ≥10% Isolates)	Total No. of O-Types (and Types With ≥10% Isolates)	Number Isolates with Yersiniabactin	ICE*Kp* ≥10% Isolates	Median No. of Virulence Determinants per Genome (Range)	Most Frequently Observed ESBLs (≥10% Isolates)	Most Frequently Observed Carbapenemases (≥10% Isolates)
11	1247	50	Asia, (49.87%);	China (456; 36.56%)	28 (KL64, 27.9%;	8 (O2v1, 46.83%;	1045 (83.80%)	8 (ICE*Kp*3, 52.34%;	1 (0–5)	CTX-M-15-523 (41.9%);	KPC-2-605 (48.5%);
			Europe, (28.46%);	Spain (149; 11.94%)	KL24, 16.4%;	O2v2, 20.16%;		ICE*Kp*4, 31.1%)		CTX-M-65-360 (28.9%)	NDM-1-188 (15.1%);
			South America, (12.34%)		KL105, 16.2%;	OL101, 13.24%)					OXA-48-156 (12.5%)
					KL47, 14.8%)						
16	957	40	Asia, (48.90%);	Thailand (334, 34.90%);	10 (KL51, 79.31%)	6 (O3b, 92.58%)	605 (63.21%)	7 (ICE*Kp*3, 48.06%;	0.71 (0–5)	CTX-M-15-821 (85.78%);	OXA-232-366 (38.24%);
			North America, (21.52%);	United States (198; 20.69%)				ICE*Kp*4, 12.43%)			NDM-1-344 (34.90%)
			Europe, (14.42%)								

## Data Availability

Not applicable here.

## Supplementary Materials

The following supporting information can be downloaded at: https://www.mdpi.com/article/10.3390/pathogens11121394/s1, Table S1: Antimicrobial resistance (AMR) genes identified in the ST16 genomes of *Klebsiella pneumoniae*; Table S2: Kleborate results for typing *Klebsiella pneumoniae* ST16 genomes; Table S3: Metal resistance genes identified in *Klebsiella pneumoniae* ST16 genomes.
